# Temporal quantification of mating system parameters in a coastal Douglas-fir seed orchard under manipulated pollination environment

**DOI:** 10.1038/s41598-018-30041-4

**Published:** 2018-08-02

**Authors:** Jiayin Song, Blaise Ratcliffe, Tony Kess, Ben S. Lai, Jiří Korecký, Yousry A. El-Kassaby

**Affiliations:** 1 Department of Forest and Conservation Sciences, Faculty of Forestry, University of British Columbia, Vancouver, BC V6T 1Z4 Canada; 2 Department of Integrative Biology, College of Biological Science, University of Guelph, Guelph, ON N1G 2W1 Canada; 3 Faculty of Forestry and Wood Sciences, Czech University of Life Sciences, 165 21 Praha, Czech Republic

## Abstract

Seed orchards main function is delivering breeding programs’ gains in the form of genetically improved seedlings. They are unique experimental populations, perfectly suited for studying various pollination environments (natural or otherwise), affecting their mating system parameters. Here, under different pollination environment (natural and intrusive (pollen augmentation and/or bloom-delay)), the mating system of a second generation, wind-pollinated, coastal Douglas-fir (*Pseudotsuga menziesii* var. *menziesii* (Mirb.) Franco) seed orchard was evaluated over four years. Using DNA microsatellite markers and bulk seed samples, we conducted pedigree reconstruction to assign each seed’s male and female parents, followed by determining the extent of pollen contamination (external gene flow), selfing rate, and, parental gametic contribution for each year. Overall, external pollen contamination rates ranged between 10 and 28%, selfing rate varied between 12 and 17%, and 80% of the seed crops were produced by 37–64% of the orchard’s parents. Pollination environment and seed crop size substantially influenced the observed results, particularly for small crops as pollen contamination was high in natural (28%) vs. intrusive pollination (10%). Generally, irrespective of the crop size, seed produced under natural pollination had higher pollen contamination, confirming the role of pollination environment manipulation in improving seed crops’ genetic quality.

## Introduction

Tree selective breeding programs are designed to increase the economic values of future forests through planting superior stock with the highest genetic gain and diversity^1^. Seed orchards are of vital importance as they represent the vehicles connecting breeding and silviculture activities through packaging gain and diversity in their genetically improved seed crops. In order to fulfil this role, seed orchards are expected to function as closed, perfect panmictic populations^2^, an ideal scenario that is hardly met due to the commonly observed variation in reproductive success and phenology among parents as well as external gene flow (pollen contamination) from the ambient environment^1,3,4^. These factors could lead to reducing the genetic worth and diversity as well as harbouring higher levels of inbreeding in the resulting seed crops^5–7^. Pollen contamination has a drastic negative impact on both growth and adaptation reaching an estimated annual economic losses exceeding 9.4 million Canadian dollars for British Columbia’s advanced generations Douglas-fir seed orchards^8^.

To mitigate the negative effects of seed orchards’ deviation from panmixia, intrusive practices affecting pollination environment such as bloom-delay (a.k.a., overhead cooling)^9^ and supplemental-mass-pollination (SMP)^10^ were devised and implemented to enhance seed crops genetic quality. Bloom-delay, originally devised as fruit species frost-protection system^11^, has also been effective in slowing within seed orchards’ heat-sum accumulation relative to the ambient environment, thus delaying their reproductive development and acts as temporal isolation from the extraneous pollen^3,12^. An added feature of the bloom-delay treatment is compacting the reproductive phenology among seed orchard’s parents as heat-sum accumulation after treatment cessation is accelerated and hence speeds reproductive development leading to panmixia improvement^13^. SMP, the application of viable pollen from selected donors to un-isolated female strobili has been successful in reducing selfing and pollen contamination^3^, harmonizing reproductive output differences among orchard’s parents and achieving increased seed yields^11,14–17^.

The availability of highly polymorphic molecular markers such as single sequence repeats (SSRs) and pedigree reconstruction made it feasible to accurately evaluate the effectiveness of seed orchard’s pollination environment manipulation practices through direct assessments of parental reproductive success and pollen contamination^4,5^. SSRs have been extensively used to study seed orchards mating patterns^5–7,18–20^ as they offer increased diagnostic power as compared to the first used allozyme markers^3,11,21,22^.

Here, we represent a continuation of a multi-year pollination dynamics assessment of a coastal Douglas-fir (*Pseudotsuga menziesii*) seed orchard^5–7^. Throughout the four studied years, this orchard’s pollination environment has been manipulated using combinations of bloom-delay and/or supplemental-mass-pollination under variable seed crop yields. Level of pollen contamination, self-fertilization, as well as parental gametic contribution are reported and compared across years to evaluate the pattern of gene flow and parental reproductive success under different conditions. To our knowledge, this is the first temporal variation study of Douglas-fir seed orchard pollination dynamics while considering both natural and intrusive pollination practices and seed crop sizes variability.

## Results

The pedigree reconstruction successfully identified the maternal and paternal parents of the fingerprinted seeds and most importantly, identified those pollinated by outside sources (i.e., external gene flow/contamination), including those from SMP external pollen donors. The contamination rate varied widely among the studied years and ranged between 10% for 2005 where the two intrusive pollination environment manipulation treatments (bloom-delay and SMP) were concurrently implemented^3^, and 28% for 2008, which was developed under natural pollination (Table 1). Years with SMP treatment alone (2007^4^ and 2009^6^) produced intermediate contamination rates (13–18%) between those of the concurrent pollination environment manipulation treatments (10%)^5^ and natural pollination (28%). Furthermore, the difference between 2007^4^ and 2009^6^ could be a reflection of the amount of SMP efforts dedicated to small verses large crops and may highlight SMP ineffectiveness during years with large crops (Table 1). It is interesting to note that the contamination rate for all intrusive pollination environment manipulation treatments^3,4,6^ (10–18%) were lower than that observed for natural pollination (28%).

**Table 1:** Temporal quantification of a Douglas-fir seed orchard’s mating system parameters (gametic contribution and gene flow, overall and true selfing rates) as affected by pollination environment manipulation along with yearly seed crop size, parental composition, and number of supplemental-mass-pollination (SMP) donors.

Year	80% Gametic Contribution			Gene Flow (%)	Overall Selfing (%)	True Selfing^a^ (%)	Crop Size (kg)	Pollination Treatment	Parental Number	Seeds Number	SMP Donors Number^b^
	♀ (%)	♂ (%)	♀ + ♂ (%)								
2005^c^	23	45	37	10	15	17	3.5	Bloom delay & SMP	49	801	16 (4)
2007^d^	39	55	52	13	12	14	19.3	SMP	57	402	23 (2)
2008^e^	57	59	64	28	16	22	2.4	Natural	58	489	N/A
2009^f^	39	45	49	18	17	21	68.8	SMP	66	207	14 (0)

^a^Selfing rate estimated within the seed orchard population after excluding those resulted from gene flow. ^b^Number in brackets indicates the number of SMP external pollen donors. ^c^Lai, B. S. *et al*.^5^. ^d^Korecký & El-Kassaby^6^, ^e^present study. ^f^Kess & El-Kassaby^7^.

Since every successful contamination event is an outcrossing event^23^, the true selfing rate should then be estimated after the exclusion of matings resulting from external gene flow. True selfing rates were consistently higher than the overall selfing rates (i.e., those based on the entire seed samples), confirming the role of contamination in under-estimating the effective selfing (i.e., inflating outcrossing rates) in the seed crop (Table 1). It is noteworthy that the difference between the true and overall selfing rates is higher when contamination is high (2% for 2005^3^ and 2007^4^ and 4% for 2009^6^ vs. 6% for 2008) (Table 1). True selfing rates varied across the studied years and ranged between 14 (2007^4^, modest size crop and SMP treatment) and 22% (2008, small size crop and natural pollination environment) (Table 1).

Considering the commonly observed “80:20” rule of percent gametes to percent parents contributing 80% of the gametes as the benchmark^24^, the parental gametic contribution fluctuated over the studied years and ranged from 37 to 64% for 2005^3^ and 2008, respectively (Table 1). The parental gametic contribution is the result of both maternal and paternal gametic contributions to the resultant seed crops (note: maternal and paternal gametic contributions are not additive). Maternal and paternal gametic contributions also varied over the years and ranged between 23 (2005)^3^ and 57 (2008), and 45 (2005 and 2009)^3,6^ and 59% (2008), respectively (Table 1). It is important to note that over the studied four years, none of the maternal or paternal and their combined parental contributions was lower than 20%, indicating that the “80:20” rule did not apply to this orchard. So, the observed distortion (i.e., deviation from panmixia (equal contribution)) is not that drastic and the seed crops are reasonable representation of the parental population.

## Discussion

Studying pollination dynamics in seed orchards is a daunting task^1,4^. Theoretically, seed orchard populations are expected to function as perfect Mendelian populations meeting Hardy-Weinberg expectations^25^. However, this idealistic scenario is rarely met mainly due to: (1) parental inherent fertility and reproductive phenology variation^26,27^, (2) uncontrollable external gene flow^28^, (3) yearly cone/seed crop fluctuations^26^ (e.g., bumper crops), (4) environmental conditions prior and during the pollination season^13^, and (5) pollination environment manipulation practices^12,29^. These factors act individually and in concert creating variable pollination dynamics across the pollination season and among years. The present study presents a complex situation where all these factors are interacting and effectively influencing the pollination dynamics of the studied Douglas-fir seed orchard.

External gene flow estimates varied among the studied years with a peak value of 28% in 2008. It should be noted that in 2008 pollination was under natural conditions (i.e., no pollination environment manipulation treatments) and, more importantly, this year has the smallest seed crop among the studied four years (2.4 kg) (Table 1). The combination of natural pollination and small cone crop created an ideal scenario for external pollen to successfully contribute to the resulting seed crop. In the Saanich Peninsula (southern Vancouver Island, British Columbia) where the seed orchard is located, Douglas-fir reproductive bud development under natural conditions is characterised by a protracted pollination season^13^. Since the orchard population is composed of individuals and their descendants selected from different locations within the orchard’s target planting area (i.e., seed planning zone^30^), then it is expected that temporal reproductive phenology development variation exists reflecting adaptation to their original location. A protracted pollination season with temporally overlapping smaller reproductively active subpopulations of a small cone crop do not produce enough pollen load to saturate receptive female strobili, hence the high contamination rate. The high external gene flow of 2008 (28%) ought to be contrasted with that of 2005^3^ (10%), another year of small seed crop (3.5 kg) (Table 1). The pollination environment of 2005 however, received both bloom-delay and SMP treatments, thus while within orchard pollen load is low, the internal reproductive phenology had been synchronized by the bloom-delay treatment^19^, and the within orchard pollen load was augmented by SMP^16^. Smaller crops tend to concentrate SMP efforts over fewer fecund individuals, thus effectively competing with the background ambient pollen. External gene flow estimates of 2007^4^ and 2009^6^ with their natural pollination environment (i.e., protracted reproductive activity) and SMP treatment produced somewhat contrasting results (13 vs. 18%) (Table 1). Following the within orchard pollen load argument presented above, the seed crop of 2009^6^ (68.8 kg) should have less external gene flow than 2007^4^ (19.3 kg). The obvious explanation for this discrepancy is the ineffectiveness of SMP in larger crops with high within orchard pollen load, a situation that calls for the strategic implementation of this practice (see first-pollination primacy below). The observed external gene flow estimates presented in this study are in line with that reported for another open-pollinated Douglas-fir orchard located in the same area of southern Vancouver Island in which pollen contamination rate of 26% was reported under natural pollination environment^8^. These external gene flow estimates are common when orchards are located within the target species’ natural range. Higher external gene flow estimates were reported in other orchards with seed crops developed under natural conditions (e.g., Douglas-fir orchards located in Western Oregon, USA (23–41%)^31,32^ and Scots pine orchard located in northern Sweden (21–36%)^28^). The negative impact of external gene flow is two-fold: (1) reducing the seed crop’s genetic worth, and (2) maladaptation if the orchard is located outside its seed planning zone^33^. In the case of the studied orchard, only genetic worth loss is expected and ranged between 7.2 and 17.6% for 2008 and 2007^4^, respectively (estimated as: (((orchard parental genetic worth weighted average) − (orchard parental genetic worth weighted average with zero value for contaminant pollen))/(orchard parental genetic worth weighted average)) × 100). Finally, it has been argued that external gene flow has a beneficial role through increasing the genetic diversity^34^; however, this is a tenuous argument considering the extent of genetic diversity in conifers^35^.

Interpreting selfing rate results is more elaborate as selfing is affected by several factors including; (1) rate of gene flow, (2) first-pollination primacy^36^, (3) SMP effectiveness, and (4) seed crop size (i.e., within orchard pollen load). Successful contamination events are in fact outcrossing matings as the pollen donors are not members of the seed orchards population^3^. Thus, it is of great importance to differentiate between overall and true selfing rates. Overall selfing is the proportion of selfed seed present in the sample of fingerprinted seed, whereas true selfing is the proportion of selfed seed after excluding external gene flow matings. Without exception, the true selfing rate must be higher than the overall selfing rate. Across the four studied years, the true selfing rate was higher than its biased overall counterpart confirming the role of external gene flow on the apparent selfing rate estimation (Table 1). It is interesting to note that 2008, the year with the highest external gene flow (28%), produced high overall (16%) and true (22%) selfing rates, a situation, at first glance, contradicts the above-mentioned external gene flow-selfing argument. Under natural pollination (no bloom-delay and SMP) and low seed crop, the orchard’s internal pollen load is low, thus first-pollination primacy must have played an important role where selfed pollen had greater chance in fertilization than unrelated pollen. Indeed, first-pollination-primacy was experimentally proven to play a significant role in Douglas-fir successful fertilization with first pollen outcompeting subsequent pollinations (“first-on, first-in”)^37^. Additional observation related to the selfing rates reported in the present study is their tendency to be generally higher than those reported from similar studies using allozyme markers, highlighting the increased discrimination power of SSRs^3,4,11,21,22^. The immediate impact of selfing is reduced seed set. In fact, an inverse linear relationship between inbreeding levels and seed set was reported in Douglas-fir (i.e., the higher the inbreeding, the lower the viable seed set)^38^. Interestingly, Woods and Heaman^38^ observed reduced seed set with lower inbreeding levels produced from matings among relatives, indicating that while consanguineous matings do not impact seed set as selfing, they are capable of producing viable seed. These seeds often succeed in producing seedlings in non-competitive environment such as container stock seedlings^39,40^, thus resulting in seedlings with reduced survival chance in competitive environments. Among the three years with SMP treatment (2005, 2007, and 2009)^3,4,6^, the observed selfing rate was negatively correlated with the number of pollen donors included in SMP mixes (Table 1). The lowest percentage of true selfing (2007^4^: 14%) is associated with the largest number of pollen donors (25) and conversely, the highest selfing rate (2009^6^: 21%) is associated with the fewest pollen donors (14) (Table 1). This observation emphasizes the importance of the number of pollen donors for increasing SMP effectiveness, in addition to other key factors such as timing, number, viability, and method of pollen application^14,16^.

The main objective of selective breeding programs is creating allelic frequencies differences between the breeding and selected populations for the attributes of interest^41^, while marinating comparable diversity level as those present in the base population^42,43^. Thus, the allelic frequencies of the seed orchard’s parental population (selected individuals) are expected to be maintained in the seed crops, so genetic gain can be captured. This situation can only be attained if the seed orchard population is in Hardy-Weinberg equilibrium, therefore the parental gametic contribution to seed crops is of substantial importance. In many seed orchards, parental fertility variation produced a common scenario where 20% of the parental population contribute 80% of the seed crops^24^. In the present study, the maternal, paternal, and combined parental gametic contribution differed between sexes and varied among years (Table 1). Generally, paternal (range: 45–59) exceeded maternal (range: 23–57) contribution and parental contribution ranged between 37 and 64%, values exceeding that of the commonly observed “80:20” rule. It is interesting to note that the two years with medium (2007^4^: 19.3 kg) and large (2009^6^: 68:8 kg) seed crops produced a scenario where 80% of the gametes were contributed by approximately 50% of the parents while the small crops (2005^3^: 3.5 kg and 2008: 2.4 kg) resulted in a wide parental contribution difference (Table 1). SMP was the pollination environment manipulation treatment for the medium and large size crops, thus increasing the effectiveness of SMP through multiple and direct applications of viable pollen from low reproductive output parents during strobili peak receptivity could be considered as an option to improving parental balance. Seed orchards parental populations’ fertility variation is unpredictable as the parental contribution of each year is different^26^, thus creating a challenging situation for predicting which parents should be included in the SMP mixes. Harvesting seed orchards crops on parental basis is a recommended option for creating customized seed crops with closer situation to Hardy-Weinberg expectations. Additionally, when the demand on seed is modest, bulking of multiple years’ crop could alleviate this deviation too.

Quantification of mating system parameters in seed orchard populations is a complicated endeavour as it is affected by multiple interacting factors; namely, parental composition, the annual environmental conditions prior and during the pollination season, extend of external gene flow, and the effectiveness of the implemented pollination environment manipulation treatments. The present multi-year study yielded an average external gene flow (pollen contamination) rate of 14% under combinations of pollination environment manipulation treatments (bloom-delay and/or SMP), and higher external gene flow (28%) under natural pollination condition. SMP effectiveness was somewhat limited under high within orchard pollen load (i.e., large crops), multiple application of pollen from low reproductive output parents is recommended for adjusting parental fertility variation. Generally, selfing rate was higher than previously reported and this is attributable to the increased SSRs discrimination power. Comparing the same seed orchard’s performance over multiple years and under different pollination environment treatment provides a general guideline for the role of these treatments on the genetic quality of seed crops. Finally, it should be stated that while the study did not follow the classical factorial experiment where pollination environment manipulation treatments were implemented in all possible combinations, the present study was informative and provided useful information on the impact of pollination environment manipulation on mating system parameters.

## Methods

### Study Seed Orchard

A second generation, clonal coastal Douglas-fir (*Pseudotsuga menziesii* var. *menziesii* (Mirb.) Franco) seed orchard established in 1990 provided the material for this investigation. It is located in the Saanich Peninsula, Vancouver Island, British Columbia, Canada (lat. 48°35′N, long. 123°24′W, elev. 50 m) and planted following the permutated neighbourhood design^44^. The orchard parental population is in constant change as lower breeding value parents are replaced by better ones (Table 1). The pollination environment of the seed orchard was manipulated using different combinations of bloom-delay^9^ and/or supplemental-mass-pollination^10^ (SMP) over the study period (Table 1). For each year, a random sample of bulk seed and vegetative buds representing the seed and parental populations, respectively, were collected for estimating the mating system genetic parameters.

### DNA Extraction and Fingerprinting

The bulk seed samples were germinated following stratification^45^ (Table 1). Germinating seeds were dissected to separate the diploid embryo from the maternal haploid megagametophyte and DNA was extracted for all parental bud tissues and embryo-megagametophyte pairs following Doyle and Doyle^46^. Polymerase chain reaction (PCR) was employed for DNA amplification for the utilized microsatellite markers (see Lai, B. S. *et al*.^5^, Korecký & El-Kassaby^6^, Supplementary Table 1, and Kess & El-Kassaby^7^ for 2005, 2007, 2008, and 2009, respectively). PCR conditions and scoring method are listed in Lai, B. S. *et al*.^5^.

### Parentage Analysis, Pedigree Reconstruction and gene flow determination

Parentage was assigned to each offspring (seed) in a two-step method using CERVUS 3.0.3^47^ (for details, see Lai, B. S. *et al*.^5^, Korecký & El-Kassaby^6^, Kess & El-Kassaby^7^). After pedigree reconstructing of each year’s seed crop, the maternal, paternal, and overall gametic contribution was calculated based on the number of offspring each parent produced as maternal, paternal, and parental (combined).

## Electronic supplementary material

Summary of allele frequency analysis for microsatellite marker evaluation
