# Supplementary material for: Temporal quantification of mating system parameters in a coastal Douglas-fir seed orchard under manipulated pollination environment
Source: Sci Rep. 2018 Aug 2;8:11593. doi: 10.1038/s41598-018-30041-4 (PMC6072752; doi:10.1038/s41598-018-30041-4)
Supplement: Supplementary file 1 — Summary of allele frequency analysis for microsatellite marker evaluation [file 41598_2018_30041_MOESM1_ESM.pdf]

Temporal quantification of mating system parameters in a coastal Douglas-fir seed orchard under manipulated pollination environment

Jiayin Song[1], Blaise Ratcliffe[1], Tony Kess[2], Ben S. Lai[1], Jiří Korecký[3], Yousry A. El-Kassaby[1*]

Table S1. Summary of allele frequency analysis for microsatellite marker evaluation.

**Locus**	**PIC[a]**	**NE - PP**
Pm_OSU2C2	0.877	0.076
Pm_OSU2G12	0.867	0.088
Pm_OSU3D5	0.909	0.048
Pm_OSU3F1	0.915	0.043
Pm_OSU3G9	0.861	0.092
Pm_OSU4G2	0.840	0.119
Mean ± SD	0.878 ± 0.026	1.5×10^{-7} [b]

[a]Polymorphic information index (Botstein, D., White, R. L., Skolnick, M. & Davis, R. W. Construction of a genetic linkage map in man using restriction fragment length polymorphisms. *Am. J. Human Genet.* **32**, 314-331 (1980)).
[b]Combined non-exclusion probability across the set of loci for parent pair (SD, standard deviation).
